# Estimating the accuracy of geographical imputation

**DOI:** 10.1186/1476-072X-7-3

**Published:** 2008-01-23

**Authors:** Kevin A Henry, Francis P Boscoe

**Affiliations:** 1 New Jersey Department of Health & Senior Services, Cancer Epidemiology Services, New Jersey State Cancer Registry, Trenton, New Jersey, USA; 2 New York State Cancer Registry, New York State Department of Health, Albany, New York, USA

## Abstract

**Background:**

To reduce the number of non-geocoded cases researchers and organizations sometimes include cases geocoded to postal code centroids along with cases geocoded with the greater precision of a full street address. Some analysts then use the postal code to assign information to the cases from finer-level geographies such as a census tract. Assignment is commonly completed using either a postal centroid or by a geographical imputation method which assigns a location by using both the demographic characteristics of the case and the population characteristics of the postal delivery area. To date no systematic evaluation of geographical imputation methods ("geo-imputation") has been completed. The objective of this study was to determine the accuracy of census tract assignment using geo-imputation.

**Methods:**

Using a large dataset of breast, prostate and colorectal cancer cases reported to the New Jersey Cancer Registry, we determined how often cases were assigned to the correct census tract using alternate strategies of demographic based geo-imputation, and using assignments obtained from postal code centroids. Assignment accuracy was measured by comparing the tract assigned with the tract originally identified from the full street address.

**Results:**

Assigning cases to census tracts using the race/ethnicity population distribution within a postal code resulted in more correctly assigned cases than when using postal code centroids. The addition of age characteristics increased the match rates even further. Match rates were highly dependent on both the geographic distribution of race/ethnicity groups and population density.

**Conclusion:**

Geo-imputation appears to offer some advantages and no serious drawbacks as compared with the alternative of assigning cases to census tracts based on postal code centroids. For a specific analysis, researchers will still need to consider the potential impact of geocoding quality on their results and evaluate the possibility that it might introduce geographical bias.

## Background

The process of assigning geographic information based on a street address, commonly referred to as geocoding, is increasingly being used in health research to assess geographic clustering or associations between health outcomes and area-based socioeconomic and/or environmental characteristics [1-4]. Geocoding is not an error-free process and inevitably some records will be imprecisely coded or not coded at all. Researchers exercise options to exclude such cases, or to include them and assign locations with a lower level of spatial precision. Rather than ignore this problem as has been done in the past, researchers are becoming more aware that either strategy may lead to geographic selection bias and misclassification of case attributes [3,5-9]. Oliver et al. (2005) refer to maps that incorporate geocoding bias as having 'cartographic confounding' and caution that results from spatial analysis may reflect the geographic "distribution of the available data rather than real, underlying disease patterns" [8]. Recent studies have shown that individual addresses not able to be geocoded to a full street address are not random and are more likely to be located in rural areas because of the disproportionate number of rural route addresses, post office box addresses, unofficial street names and streets missing from geocoding reference files [5,10-16].

To reduce the number of non-geocoded cases and thus limit potential geographic selection bias, researchers and organizations sometimes include cases geocoded to postal code centroids along with cases geocoded with the greater precision of a full street address [17-21]. Central cancer registries, for example, assign cases to census tracts based on postal ZIP code centroids when full address based geocodes are not available. This practice is encoded in the Census Tract Certainty and GIS Coordinate Quality fields in the data standards used by central cancer registries in the United States [22]. Some analysts then use the centroid locations to assign information to the cases from finer-level geographies (such as block group), even though this practice may fall prey to the ecologic fallacy. In the United States, postal ZIP codes in urban and suburban areas are typically larger than census-level geographies such as census tracts (averaging 4,000 people) and census block groups (averaging 1,500 people). Assigning a tract or block group based on the postal ZIP code centroid can potentially lead to overestimation of small area case counts (if multiple cases are assigned to the same ZIP code centroid), to misclassification of the area-based variables assigned, and/or to bias in specific kinds of spatial analysis such as estimates of clustering.

To minimize problems that might be associated with using postal ZIP code centroids, researchers have devised probabilistic methods to assign a reasonable location by using both the demographic characteristics of the case and the population characteristics of the postal delivery area in which it falls. If, for example, a researcher was attempting to assign a census tract for an Asian case with only information about his/her postal ZIP code, the most likely tract would be the one with the largest Asian population. To describe this process of assigning information about a finer geographic unit based on information from one or more coarser geographical units we use the term 'geographical imputation' or 'geo-imputation'. Imputation techniques are well established as a general useful method for the analysis of data with missing values and are included in many popular statistical packages [23,24]. There is, however, a dearth of literature covering the accuracy and usefulness of geographical imputation.

Geographical imputation has been applied in numerous studies to assign geographic locations. Sheehan et al. (2004) assigned non-geocoded Massachusetts breast cancer cases to census tracts within their town boundaries. They related town boundaries to the census tract boundaries and randomly assigned tracts to cases based on the proportion of the town's female population accounted for by each census tract [25,26]. Klassen et al. (2006) assigned Maryland prostate cancer cases not geocoded to a full address to census block centroids using imputation based on the race, age and gender specific population distributions within their ZIP codes [27,28]. Huang et al. (2007) assigned random locations within imputed census blocks for Los Angeles cancer cases used in geographic analysis of cancer survival [29]. Boscoe (2007) provides hypothetical examples of how geographical imputation can be used to assign a cancer case to an area (e.g. census tract or block group) to give the case latitude and longitude coordinates within the tolerances of available information on geography (e.g. postal ZIP code or county) and demography (e.g. race).

Despite these examples, to date no systematic evaluation has been completed to assess whether geo-imputation is preferable to coding tracts based on postal ZIP code centroids. In this study, we evaluate the accuracy of census tract assignments, by using a large dataset of breast, prostate and colorectal cancer cases reported to the New Jersey Cancer Registry. We particularly focus on a common situation for researchers and central cancer registries where we have information about the patient's age, race/ethnicity, and ZIP code. Our goal is to determine the accuracy of census tract assignment using geo-imputation, and whether geo-imputation provides more accurate assignments than can be obtained from a postal ZIP code centroid, either the geographical centroid or a population-weighted centroid. This paper takes only a first step toward assessing the advantages and disadvantages of geo-imputation. We address the specific question of accuracy of census tract assignments on the basis of population distributions within ZIP codes. Under alternative strategies of imputation, how often is the case assigned to the correct census tract?

## Methods

### Data sources and study population

The case data used for this analysis included 99,502 New Jersey residents diagnosed with breast, prostate or colorectal cancer between 2000 and 2004. Exclusions for this subset included cases with invalid ZIP codes or PO boxes, cases missing age, and/or race, cases less than 21 years of age, and cases falling within ZIP code areas with only one census tract (N = 2578). The final subset (N = 96,924) included 37,267 (38.4%) breast cancer cases, 33,244 (34.3%) prostate cancer cases, and 26,413 (27.2%) colorectal cases. This subset represents 38% of all cancer cases reported to the NJSCR for this period.

All cases were geocoded to their address at time of diagnosis by the New Jersey State Cancer Registry (NJSCR). The NJSCR is a population-based incidence registry that covers all cancer cases diagnosed in New Jersey since 1979. The NJSCR employs both automated and interactive geocoding. The automated geocoding system uses Integrity Geolocator software and the most recent street file available from Tele Atlas [30,31]. Interactive geocoding was completed using ArcGIS 9 software and Tele Atlas street files [31,32]. Approximately 1% of the cases included in this study were manually geocoded. Cases were assigned to their corresponding year 2000 U.S. census tracts using the street segment of their address.

The NJSCR case variables used in this study included age at diagnosis and race/ethnicity. Race and age are reported to the NJSCR for all newly diagnosed cancer cases by reporting agencies such has hospitals, physicians, and clinical laboratories. We grouped race/ethnicity as non-Hispanic white (White), Non-Hispanic black (Black), Asian/ Pacific Islanders (API), and Hispanic. Individuals who were not coded as one of these race/ethnicity groups were excluded (N = 1621). Age was grouped into four categories (21–49, 50–64, 65–84, >85). Using the known census tract for each case we additionally assigned cases into population density quintiles [33]. Table 1 provides the case counts for the different subsets used in this study.

**Table 1:** Summary of population subsets used in study.

Population Subset	Cases
Total Population	95,303
	
Non-Hispanic White	75,577
Asian	2,235
Non-Hispanic Black	11,123
Hispanic	6,368
	
Non-Hispanic White	
20–49	8,597
50–64	22,191
65–84	39,388
>85	5,401
	
Asian	
20–49	563
50–64	852
65–84	773
>85	47
	
Non-Hispanic Black	
20–49	1,502
50–64	4,157
65–84	5,032
>85	432
	
Hispanic	
20–49	1,269
50–64	2,252
65–84	2,605
>85	242

Source: Data from the New Jersey State Cancer Registry, New Jersey Department of Health and Senior Services, 2005

### Creation of census tract populations

Both the U.S. postal ZIP code and census tract boundaries were based on the 2007 Tele Atlas Dynamap data [34,35]. U.S. postal ZIP codes are 5 digit numeric values that correspond to address groups or delivery routes. The postal ZIP code boundaries are a cartographic representation derived from the most recent 5-digit postal delivery information available from the United States Postal Service. New Jersey has approximately 735 ZIP codes, of which 557 are considered general use ('non unique'), 139 are PO Boxes, and 39 represent facilities, businesses, or government agencies that receive large volumes of mail. We included only cases having general use ZIP codes.

**Figure 1:**
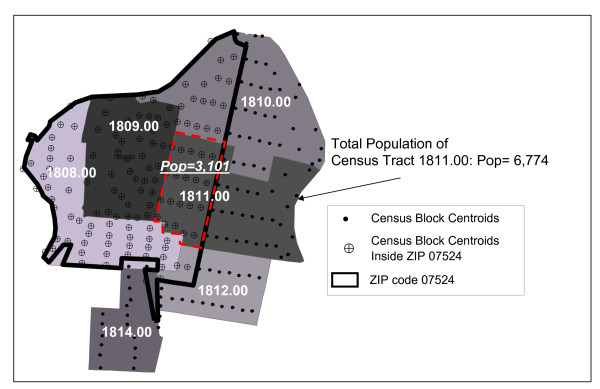
**Census block centroid populations are used to calculate the proportion of census tract populations which fall within the boundaries of ZIP codes**. For example, the portion of census tract 1811.00 within ZIP code 07524 receives only 3,101 individuals of the total census tract population of 6,774.

Because some census tracts overlap postal ZIP code boundaries, we used census block centroid locations to recalculate the portion of the tract population falling within each ZIP code [36]. We used a point in polygon operation in ArcGIS 9.1 software to assign each census block a ZIP code and then aggregated the data based on unique county, census tract, and ZIP code identifiers (Figure 1) [32]. Figure 1 provides an example of how populations were assigned to census tracts using census block centroids. This figure illustrates how within ZIP code 07524, tract 1811.00 receives only a portion of the total census tract population based on the census blocks falling within its boundary. Using data from the 2000 U.S. Census Short form (SF1), for each ZIP code we calculated the percent of the population in each tract by race/ethnicity alone and by race/ethnicity for the four defined age groups. The following race/ethnicity and age census populations were included: total population (P001001), non-Hispanic White (P004005, PCT12I), non-Hispanic Asian (P004008, PCT12L), non-Hispanic Black (P004006, PCT12J), and Hispanic (P004002, PCT12H) [33].

### Geo-imputation

**Figure 2:**
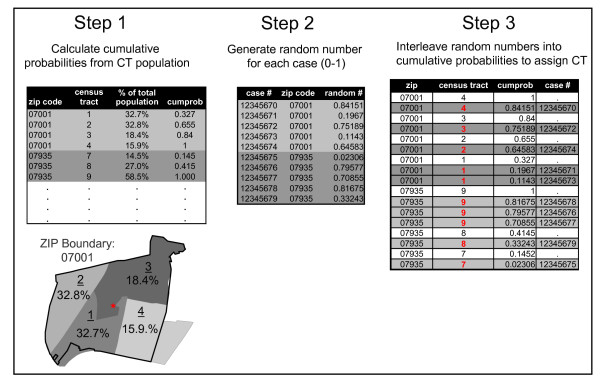
Procedures used for geo-imputation.

The geo-imputation method used for this study assigns a census tract to a case based on the fraction of the population from each tract located within the bounds of each postal ZIP code. Tracts having a greater fraction of the population have a higher probability of being assigned to cases. Our imputation procedure required three steps (Figure 2). First, using the tract population fractions for each population subgroup in each postal ZIP code, we calculated cumulative probabilities. Second, we assigned a random number between 0–1 to each case using the SAS function 'ranuni'. Finally, we interleaved the random numbers into the census tract cumulative probabilities for each ZIP code and assigned each case a census tract based on where the random number fell within the range of cumulative probabilities. Figure 2 provides a hypothetical example for imputing cases with ZIP codes 07001 and 07935. All geo-imputation steps were completed in SAS v. 9.1 [37].

To appraise the accuracy of assignment of census tracts for the several subsets (race/ethnicity and age), we employed a resampling method, executing steps 2 and 3 of the procedure 1000 times for the following cumulative probabilities: 1) total population, 2) race/ethnicity, and 3) race/ethnicity and age. After each iteration, we compared the census tract assigned by the program with the tract originally identified from the full street address. Were they the same? For each subset and set of iterations, we report mean, minimum and maximum percentages matched and display their properties as box-and-whisker plots.

Additionally, we estimated the accuracy of assigning census tracts based on both the geographic-centered and population-weighted postal ZIP code centroids by comparing these with the known census tracts [34,38]. Geographic centroids were constructed as the center of the bounding rectangle for the zip polygon, and latitude-longitude coordinates for the population-weighted centroid were calculated as the mean of coordinates of centroids of the census blocks encompassed, weighted by the numbers of persons in the respective blocks. As a further alternative, tracts were assigned to cases on the assumption that every tract in the postal ZIP code had the same chance of being selected (random assignment).

## Results

On the basis of match rates shown in Table 2, geo-imputation using more specific covariates more often assigned cases to the correct census tracts. Some methods of assignment produced higher rates than others. For the entire study population, cases assigned to census tracts based on geographic centroids had correct matches 20.7% of the time compared with 25.9% when using population weighted centroids, and 27.7% when geo-imputation was based on a distribution of population by race/ethnicity-age (Table 2).

**Table 2:** Summary of results from different census tract assignment methods (ZIP code centroids and geo-imputation).

Population	Cases	Random^a^	Geographic Centroid^b^	Population Centroid^c^	Overall Population Distribution	Population Distribution by Race/Ethnicity	Population Distribution by Race/Ethnicity-Age
	N	Mean % (Min, Max %)	Total %	Total %	Mean % (Min, Max %)	Mean % (Min, Max %)	Mean % (Min, Max %)
All Cases	95,303	14.1 (13.5,14.7)	20.7	25.9	25.8 (25.3, 26.3)	26.7 (26.0, 27.4)	27.7 (26.5, 29.0)
Non-Hispanic White	75,577	15.0 (14.6, 15.4)	22.0	27.9	27.8 (27.4, 28.1)	28.2 (27.7, 28.7)	29.3 (28.4, 30.3)
Non-Hispanic Black	11,123	9.8 (9.0, 10.8)	15.3	16.6	16.5 (15.7, 17.4)	20.1 (18.7, 21.0)	21.3 (19.5, 23.2)
Hispanic	6,368	11.1 (10.0, 12.4)	16.3	19.2	18.8 (17.4, 20.0)	19.8 (18.7, 21.3)	20.2 (17.5, 22.7)
Asian	2,235	13.7 (11.6, 15.8)	17.3	25.4	26.0 (24.2, 28.5)	28.8 (26.4, 31.5)	28.8 (24.5, 33.5)
Age							
20–49	11,931	14.2 (13.4, 15.2)	22.0	26.5	26.5 (26.4, 27.4)	27.5 (26.4, 28.4)	27.7 (25.6, 29.5)
50–64	29,452	14.2 (13.6, 14.8)	21.1	26.4	26.2 (25.6, 26.6)	27.0 (26.2, 27.3)	27.4 (26.2, 28.6)
65–84	47,798	14.1 (13.6, 14.5)	20.3	25.4	25.6 (25.1, 26.0)	26.5 (26.1, 27.2)	27.8 (26.8, 28.8)
>85	6,122	13.9 (12.8, 15.3)	19.4	25.9	25.2 (23.9, 26.4)	25.9 (24.1, 27.4)	29.1 (26.9, 31.3)
Population Density							
<1,132	17,262	16.5 (15.9, 17.2)	29.6	29.6	30.8 (30.1, 31.2)	31.4 (30.3, 32.1)	31.8 (30.5, 33.1)
1,133–2,882	23,036	15.3 (14.9, 15.8)	24.1	29.0	30.2 (29.7, 31.0)	30.8 (30.2, 31.2)	32.4 (31.5, 33.4)
2,883–5,078	21,744	14.1 (13.4, 14.6)	19.2	27.1	25.6 (24.9, 26.2)	26.4 (26.7, 27.4)	27.8 (26.7, 28.7)
5,079–11,579	18,822	14.7 (13.9, 15.5)	17.1	26.3	24.9 (24.8, 25.3)	26.4 (25.5, 27.2)	26.8 (25.3, 28.2)
>11,579	14,439	8.6 (7.9, 9.3)	12.0	14.3	14.3 (13.6, 15.0)	15.5 (14.3, 16.2)	16.3 (14.9, 17.6)

^a^Random assignment was based on census tracts having equal probability within each postal ZIP code.

^b^Geographic postal ZIP centroids were based on the center of the bounding rectangle for each ZIP code area.

^c^Population weighted postal ZIP centroids were calculated as the mean of the census block centroid coordinates weighted by the number of persons per block.

Source: Data from the New Jersey State Cancer Registry, New Jersey Department of Health and Senior Services, 2005.

**Figure 3:**
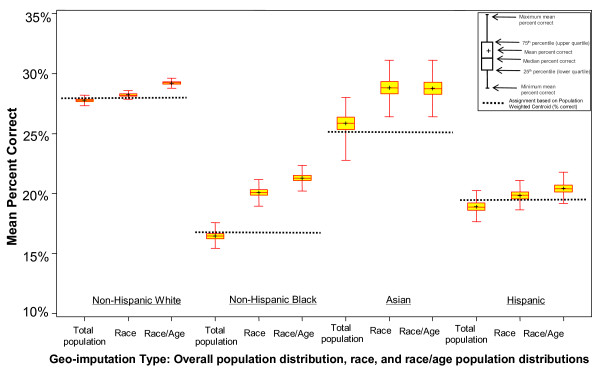
Mean percent of correct census tract matches using geo-imputation based on the overall population distribution, and population distributions by race/ethnicity, and race/ethnicity-age.

In each of the 4 race/ethnicity groups used in this study, the percentage of cases assigned to the correct census tract was always highest when census based population distributions by age and race/ethnicity were used. As the box-and-whisker plots in Figure 3 illustrate, as additional information is introduced for cases and for census tract populations, the match rates increase. Increases were most pronounced when race/ethnicity specific geo-imputations were applied to cases among Asians (66%) and non-Hispanic Blacks (31%), and least pronounced when applied to non-Hispanic Whites (28%) and Hispanics (21%). While geo-imputation based on the overall population distribution resulted in fewer tract matches than using population weighted ZIP centroids it did have more matches than assignment based on geographic ZIP centroids (Table 2, Figure 3).

**Figure 4:**
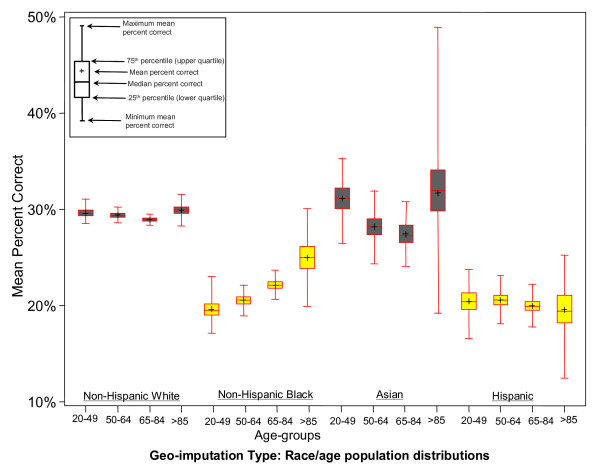
Mean percent of correct census tract matches by geo-imputation based on race/ethnicity/age groups.

The box-and-whisker plot in Figure 4 summarizes the effect of age on geo-imputation for each race/ethnicity group. For age-specific geo-imputation, the highest percentages of matches characterize the older age groups except for Hispanics (Figure 4). Non-Hispanic Blacks were the only group showing a significant increase in the percent of matches at each increment of increasing age. The wide range in minimum and maximum match values for the age specific geo-imputations is the result of small numbers. As summarized in Table 2, when these results by age groups are combined for each race/ethnicity group the overall range in minimum and maximum matches are reduced. For the race/ethnicity-age based geo-imputation, the mean percent of census tract matches were greatest for non-Hispanic whites (29.3%) and Asians (28.8%) and lowest for non-Hispanic Blacks (21.3%) and Hispanics (20.2%).

Geo-imputation match rates for the various subsets are a function of two factors: population densities across New Jersey and the geographical distributions of the groups. As summarized in Table 2, matches were consistently lowest in the most densely populated areas and increased as population density decreased. For geographic ZIP centroids, tract assignment was best in areas with the lowest population density, but match rates decreased more rapidly with increasing population density when compared with assignment based on weighted centroids. Population weighted centroids appear to provide higher match rates across the range of different population densities compared with geographic centroids.

## Discussion

From this experiment, we might rank preferences for the various strategies. Assigning cases to census tracts on the basis of postal ZIP code was most effective when we used census tract probabilities that took into account more specific covariates (e.g. race/ethnicity and age). Each addition of more specific covariate information resulted in more cases being assigned to their correct tract. Gains with introduction of more covariates is a finding consistent with the non-spatial literature of imputation [24].

Assignment based on population weighted centroids resulted in more cases being matched to their correct tract compared with assignment based on geographic centroids. Additionally, the population-weighted centroids performed better across a range of population densities, and, in suburban New Jersey, more often fell where people were actually living. Some centroids still fall into an open space occupied by an airport, a swamp, or a large factory, but they produce overall a better approximation of where people live.

The overall gain in correctly assigned tracts using race/ethnicity-age based geo-imputation varied depending on which ZIP code centroid type we compare. The gain was highest compared with assignment using geographic centroids and lowest compared with assignment using population weighted centroids. For every 1000 cases, the improved match rate from geo-imputation would allow one to assign about 70 more cases to their correct tract than assignment based on geographical ZIP centroids, and only 19 more cases compared with assignment based on population weighted centroids. Geo-imputation based on only the total population distribution produced match rates similar to those based on population-weighted centroids.

These findings raise two important points about whether it is worthwhile to implement geo-imputation methods since the gain achieved assigning census tracts was low compared with population weighed centroids. First, applying geo-imputation with more covariates did result in more census tract matches, thus likely reducing the actual amount of overestimation and misclassification. However, since the gain was small we can not conclude whether it's enough to make a significant difference reducing any bias in a typical study. Second, many organizations and cancer registries, (including the New Jersey State Cancer Registry) use geographical centroids instead of population weighted centroids because they are freely available or provided by vendors. In this situation the gain in correct tract assignments using geo-imputation compared with geographic centroids was higher, though it is still unclear whether this would make a significant difference in reducing bias in a typical study

The amount of overestimation, misclassification and/or bias geo-imputation could conceivably eliminate would vary based on the overall geocoding quality, a study areas population density and socio-demographics characteristics. And as well, would be different for a study with 12% of its cases geocoded to a ZIP code centroid compared with a study with only 1% geocoded to a ZIP code centroid, or where the majority of cases geocoded to ZIP code centroids are concentrated in one part of a study area. We plan to focus on these important issues in future research.

The ideal alternative to geo-imputation would be to improve the quality of the original address record so that the number of ungeocoded cases is too small to have an impact on a study. In Canada and other countries with highly precise postal codes, this is typically the case, as the postal code itself offers precision to a block-face or a single large apartment building [39]. Many products are already available for researchers to link Canadian postal codes to various levels of census boundaries including enumeration areas and dissemination areas. In the U.S., increased use of the ZIP +2 and +4 codes could offer large gains in locational precision, but the extra digits are not presently required by the postal service, most residents do not know them, and so they do not find their way into medical records.

Although geo-imputation does not, by any means, fully compensate for low-quality geocoding, it can make some improvement in assignments of cases to census tracts. In a well-designed project, researchers, by considering the geographical distribution of cases geocoded to a lower standard (the postal ZIP code alone), can obtain greater control over sources of bias that may affect the risk factors or clustering patterns they are trying to uncover. As we have seen, match rates are strongly associated with population density, which is in turn the number of census tracts per postal ZIP code: in urban areas there are many, while in rural areas there are few. If the majority of cases missing full address information are from rural areas, which the literature suggests is most often the situation, then geo-imputation will provide a higher match rate than if the majority of these cases were from more urban areas. In this study when race/ethnicity-age geo-imputation was used, cases in highly urban areas were only assigned to the correct tract 16.3% of the time, while cases from the most rural areas had twice as many correct tract assignments (Table 1).

Besides population density there are several other important factors that could impact the number of cases assigned to correct census tracts if these methods were applied in other U.S regions. First, researchers must consider the geographical distribution of the populations which they are using as a basis for calculating match probabilities. If certain race groups are geographically segregated then match rates will depend on their location (e.g. rural vs. urban) and the socio-demographics of the census tracts. Areas with socio-demographically homogeneous census tracts will likely have lower match rates. In our analyses, for example, the census tract match rate was lowest for non-Hispanic Blacks than other race groups because in New Jersey the majority of the Black population is geographically concentrated in urban areas.

Second, researchers should consider the geographical accuracy of the census tract and postal ZIP code boundary files that are used to perform areal interpolations. Misalignments of boundaries during the spatial operations procedures to calculate the proportion of each census tract within a postal ZIP code could result in incorrectly specified census tract populations. Lastly, researchers must consider changes in postal ZIP codes and ensure the time period for which the cases represent match the time period of the related valid postal ZIP codes. Any changes in ZIP code numbering and/or boundary reconfiguration could impact results.

Several study limitations should be considered when interpreting the present results. First, we used a point in polygon operation with census block centroids to calculate the census tract populations falling partially within ZIP codes. Numerous studies have found the point in polygon approach to over or under-estimate census tract populations when compared with techniques such as areal weighting. While the use of the point in polygon technique would likely have a neglible effect on the outcome, future work involving geographic-imputation should consider areal weighting. Additionally, studies interested in imputing more precise locations such as census block groups might consider a dasymetric mapping approach which would remove uninhabitable areas and redistribute the population. Previous research covering population interpolation, areal interpolation, and dasymetric mapping of socioeconomic data could be adapted to allow for more accurate geographical imputation [36,40-43]. Research is needed to assess which methods would be best suited.

A final limitation is the results from this study were based on a subset of known addresses and their associated geographic distribution rather than a subset of unknown addresses. Since rural areas typically have lower geocoding rates and fewer candidate census tracts per ZIP code than urban areas, in a typical statewide study, overall match rates using the different strategies of geo-imputation would likely be slightly higher than those reported in this study.

While geo-imputation does appear to offer some advantage compared with assigning census tracts based on a postal ZIP code centroid, its usefulness will hinge ultimately on studies that can determine associations between geocoding quality, spatial bias, and social bias. Ratcliffe (2004), for example, using crime data, tested what minimum number of geocodes was necessary to produce a statistically accurate map [44]. In the past, researchers have often attempted to use the smallest available geographic unit regardless of bias that would result from incomplete data. Where quality of geocoding is low and introduces bias, it would be advantageous instead to resort to a larger spatial unit of analysis, to include a larger proportion of the desired sample.

Further work is needed to test the extent of geocoding quality in a  variety of "real-world" research situations - at small scales, on  infectious diseases as well as cancers, and in contexts where we have  a good understanding of the scale and sources of clustering. Further  research is required also to test the extent to which the geographical  misclassifications (among census tracts) are likely to affect the  area-based variables imputed. Since large and costly databases like cancer  registries are designed for long-run and cumulative value, we will  need to progress in geocoding, to employ the "certainty" and "quality"  coding fields, to retain the possibility of re-assigning cases, and to  do ex post facto studies that will provide better evidence of how many  cases we can afford to exclude, or how many instances of low-quality  geocoding we can tolerate without jeopardizing a research design.

Some central cancer registries and organizations have adopted the practice of automatic assignment of cases to a census tract based on a postal ZIP code centroid whenever they do not have a more complete address. These organizations might instead find it useful to shift to the use of centroids that take population into account, or to use some form of geo-imputation. They might consider leaving the records without geographic coordinates but offering geo-imputation (e.g. to census tract level) at the time of a particular data request or research project. It is important, however, that each organization develop an assignment strategy that works best with their data, can be completed quickly, and is well documented.

As in conventional, non-spatial statistics, serious problems can arise with unusual distributions resulting from missing data or imprecisely coded data. In practice, these are rare, but the search for associations between area based socio-economic and environmental factors and disease (like the search for spatial clusters) is a search for unusual distributions. It is necessary, therefore, to develop geocoding best-practice precautions and techniques to enable accurate spatial analyses. While more research needs to be done to assess the practice of geo-imputation, this study provides an important first step toward assessing the advantages and disadvantages of geo-imputation for assigning cases to census tracts based on a postal ZIP code centroid. Until best practice precautions and techniques are developed and confirmed, researchers need to consider the impact of geocoding quality and completeness on study results and be attuned to potential geographic bias.

## Authors' contributions

The study design and question was conceived by KH and FB. KH completed data processing and subsequent analysis and wrote manuscript. FB provided suggestions and comments on the manuscript. Both authors read and approved the final manuscript.
